# Applications of Metabolomics in Forensic Toxicology and Forensic Medicine

**DOI:** 10.3390/ijms22063010

**Published:** 2021-03-16

**Authors:** Michal Szeremeta, Karolina Pietrowska, Anna Niemcunowicz-Janica, Adam Kretowski, Michal Ciborowski

**Affiliations:** 1 Department of Forensic Medicine, Medical University of Bialystok, 15-269 Bialystok, Poland; michalszeremeta@gmail.com (M.S.); anna.janica@umb.edu.pl (A.N.-J.); 2 Metabolomics Laboratory, Clinical Research Center, Medical University of Bialystok, 15-276 Bialystok, Poland; karolina.pietrowska@umb.edu.pl (K.P.); adamkretowski@wp.pl (A.K.); 3 Department of Endocrinology, Diabetology and Internal Medicine, Medical University of Bialystok,15-276 Bialystok, Poland

**Keywords:** forensic medicine, forensic toxicology, metabolomics, drugs of abuse, postmortem interval

## Abstract

Forensic toxicology and forensic medicine are unique among all other medical fields because of their essential legal impact, especially in civil and criminal cases. New high-throughput technologies, borrowed from chemistry and physics, have proven that metabolomics, the youngest of the “omics sciences”, could be one of the most powerful tools for monitoring changes in forensic disciplines. Metabolomics is a particular method that allows for the measurement of metabolic changes in a multicellular system using two different approaches: targeted and untargeted. Targeted studies are focused on a known number of defined metabolites. Untargeted metabolomics aims to capture all metabolites present in a sample. Different statistical approaches (e.g., uni- or multivariate statistics, machine learning) can be applied to extract useful and important information in both cases. This review aims to describe the role of metabolomics in forensic toxicology and in forensic medicine.

## 1. Introduction

In 2002, O. Fiehn wrote that “Metabolites are the end products of the cellular regulatory processes, and their levels can be regarded as the ultimate response of biological systems to genetic or environmental changes.” In addition to this idea, he also stated: “In parallel to the terms ‘transcriptome’ and ‘proteome’, the set of metabolites synthesized by a biological system constitute its ‘metabolome’ [1]. In 2006, J.K. Nicholson defined metabolomics simply as “The comprehensive quantitative analysis of all the metabolites of an organism or specified biological sample” [2]. Metabolomics has evolved highly over the last years and is now described as the study of metabolites using advanced high throughput analytical approaches and bioinformatics [3,4,5]. Metabolomics monitor changes in small molecules (<1500 Da) with modifications appearing in organisms in response to a specific stimulus [6]. In contrast to the other “omics sciences”, metabolomics can link both gene and environmental interactions. The metabolites present in biological systems, defined as the metabolome, include endogenously derived biochemicals like amino acids, lipids, fatty acids, steroids, carbohydrates, or vitamins. Although still developing in forensics, the metabolomics approach is already considered a helpful tool for various legal questions (e.g., estimation of the postmortem interval, PMI) [7]. Metabolomics techniques can also be useful for indicating novel biomarkers with better diagnostic performance than those already existing [8] or estimating mortality risk in global disease (e.g., acute myocardial infarction) [9]. Techniques of metabolomics and computational biology have also been used to build a quantitative forecast model for early warning of rapid death [10]. On the other hand, analyses of metabolic pathways might be successfully used to explore consumption behavior, differentiate between acute or chronic drug use, or to find the underlying mode of toxicological action in humans [11].

## 2. Analytical Techniques Used to Study the Metabolome

Metabolomics analysis can be performed using different approaches. The most comprehensive is metabolic fingerprinting, which ideally aims to measure all metabolites present in the studied sample. In reality, none of the currently used analytical techniques can measure all metabolites present in a biological sample. Therefore, a combination of several techniques to analyze the same sample is required to increase metabolome coverage. Metabolic fingerprinting, also called untargeted metabolomics analysis, can be performed using nuclear magnetic resonance (NMR) or mass spectrometry (MS) in combination with one of the separation techniques: liquid chromatography (LC), gas chromatography (GC), or capillary electrophoresis (CE). In comparison to MS, NMR has a lower sensitivity but better reproducibility. Additionally, NMR requires minimal sample preparation, which is inherently quantitative and non-destructive to the sample [12]. Depending on the separation technique hyphenated to MS, different classes of metabolites are detected. GC-MS can be used to measure volatile metabolites or metabolites that can be transformed into a volatile form [13]. CE-MS is useful for the detection of polar and ionogenic metabolites [14]. At the same time, depending on the type of the chromatography used, LC-MS can be applied to measure polar (hydrophilic interaction liquid chromatography) and non-polar (reversed-phase chromatography) metabolites [15]. In the case of untargeted studies, the use of high-resolution, usually orbitrap or time of flight (TOF), MS analyzers is required [16]. The same analytical techniques can be used for other metabolomics approaches, like metabolic profiling or target analysis of metabolites. However, in these approaches, previously specified metabolites are measured. Metabolic profiling can be performed for metabolites belonging to a particular class (e.g., fatty acids, amino acids [17]) or metabolites from the specific metabolic pathway (e.g., arachidonic acid and its cyclooxygenase (COX) and lipoxygenase (LOX) pathway metabolites) [18]. Whilst MS-based untargeted metabolomics is a semi-quantitative analysis (a study group is compared to a control group to select significantly discriminating metabolites), methods used for targeted metabolomics and metabolic profiling are usually quantitative. However, to quantify metabolites, the use of their analytical standards is required and, if possible, isotopically labelled standards are preferred. Profiling and targeted metabolomics are often used to confirm untargeted results and/or evaluate the diagnostic utility of potential biomarkers [19].

In the case of MS-based metabolic fingerprinting, extensive data treatment is required. First, samples are analyzed under the control of the quality control (QC) sample, which, if possible, should be prepared by pooling equal volumes of all studied samples. Obtained data undergo a quality assurance procedure, which aims to select reproducibly measured metabolites [20], and are filtered to keep only metabolites present in the majority of the samples [21]. Such a dataset can be forwarded to statistical analysis. In the case of GC-MS data, identification of metabolites is performed before statistical analysis, while in the case of CE-MS and LC-MS data, statistical analysis is performed before identification [22]. A pathways analysis can be performed to understand the role of significant metabolites better and interpret the obtained results. There are several tools for mapping metabolites into biochemical pathways (e.g., MassTRIX) or combining metabolomics data with other omics data on metabolic pathway map (e.g., ProMeTra) [23]. Moreover, a comprehensive metabolomics data analysis, interpretation, and integration with other omics data can be performed with a freely available on-line tool MetaboAnalyst (www.metaboanalyst.ca (accessed on 8 February 2021)) [24].

## 3. Applications of Metabolomics in Forensic Toxicology

### 3.1. Drug Abuse with Attention to New Psychoactive Substances

Drug abuse, especially new psychoactive substances (NPS), is a growing problem worldwide. Global drug-related mortality has increased by 60% in the years 2000–2015 [25]. The use of metabolomics opens the possibility of identifying some novel markers of forensic interest. The holistic profiling approach can be used to profile the range of chemical xenobiotics and their metabolites in humans, which can be referred to as the xenometabolome [26]. Detection of xenobiotic metabolites with the use of LC-MS and multivariate discriminant analysis (MDA) was first proposed by Plumb et al. [27].

Conventional methods of analysis may no longer be effective in screening NPS. In screening approaches, particularly if the parent compound itself remains undetectable in specimens, drug intake detection will be possible over one or a few unique metabolites. However, in the case of common primary metabolites for several structurally related compounds, other minor metabolites might be necessary to prove the intake of a particular illegal drug. Thanks to the enhanced resolution of MS, several algorithm-based methods such as mass defect filtering techniques have been developed in the drug metabolism field [28]. Metabolomics-related procedures present an alternative strategy for the identification of biomarkers and might be highly beneficial to provide fast response to suspected NPS consumption and aid in the overall diagnostics of drug abuse or overdose.

Metabolomics can also be used as an alternative method to detect new drug metabolites in different human specimens [29,30,31]. For example, Steuer et al. [29] analyzed urine samples using untargeted metabolomics to search for new biomarkers of gamma-hydroxybutyric acid (GHB) intake. Analysis of urine samples collected 4.5 h after GHB or placebo consumption of a randomized, double-blind, placebo-controlled crossover study in 20 men allowed for the identification of new GHB metabolites like GHB carnitine, GHB glycine, and GHB glutamate [29]. Metabolomics studies in this area were recently performed to find endogenous markers that might be able to prolong the short detection window (only up to 12 h in urine) of GHB [32]. Mollerup et al. [31] performed a retrospective analysis to identify potential markers of valproic acid in the blood. They used liquid chromatography coupled with high-resolution mass spectrometry (LC-HRMS) to identify eight potential targets for valproic acid [31]. In 2011, Shima et al. [33] used a combination of GC with time-of-flight MS and CE with tandem-MS for global and targeted analyses and proposed the endogenous compounds to be considered as potential markers of methamphetamine (MA) intoxication. Potential biomarkers related to MA-induced poisonings included 5-oxoproline, saccharic acid, uracil, 3-hydroxybutyrate, adipic acid, glucose, glucose 6-phosphate, fructose 1,6-bisphosphate, and fumarate [33]. In 2016, Nielsen et al. [34] performed an untargeted metabolomics experiment on a retrospective dataset collected for forensic toxicology routine analysis. The researchers compared whole blood samples from living Danish drivers positive for 3,4-methylenedioxymethamphetamine (MDMA) in different concentrations to negative control samples using various statistical methods. The fact that MDMA and its metabolites were positively identified by applied methods and upregulated in MDMA users proved successful overall data analysis [34].

Analysis of small molecule metabolites can be useful to examine the synthetic cannabinoids (SCs) and stimulants, which make up the most significant fraction of newly reported NPS. SCs are consumed as a legal alternative to cannabis and often allow for the passing of drug-screening tests. The development of fast screening tests for SCs, not directly focused on their chemical structure, would be highly appreciated in forensic laboratories. This strategy was developed to find markers for herbal mixtures, which act as the base for “spice” products. In the experiment prepared by Bijlsma et al. [35], saliva samples of healthy volunteers were collected at pre-dose and after smoking herbal components and analyzed by high-resolution mass spectrometry. The data, combined with statistical analysis, allowed for the discrimination of potential positives based on the analysis of two markers (scopoletin and 2-hydroxyethyl dodecylamine) identified in the herbal blends, whose ratio permitted distinguishing the herbal smokers from non-smokers [35].

### 3.2. Drug Addiction

Novel metabolomics approach connected to the drug of abuse did not only focus on biomarkers indicating acute drug consumption, but also on the identification of guide for drug addiction, the intensity of drug addiction or the interpretation of the level of intoxication. Currently, the available literature is focused on MA [36], cocaine [37], crack [38], and heroin [39]. For example, Yao et al. [37] characterized cocaine metabolism in mice and rats through LC-MS-based metabolomics analysis of urine samples. The data showed that benzoylecgonine levels were similar in both groups treated with the same dose of cocaine. However, the levels of *N*-hydroxybenzoylnorecgonine and hydroxybenzoylecgonine (the cocaine metabolites from oxidative metabolisms) differed dramatically between the two species. Structural study through precise mass analysis and LC-MS/MS fragmentation revealed that *N*-oxidation reactions including *N*-demethylation and *N*-hydroxylation were among the mouse’s metabolic routes. In contrast, extensive aryl hydroxylation reactions occurred in the rat. The differences in the oxidative metabolism of cocaine between the two species were confirmed by in vitro microsomal incubations. Chemical inhibition of P450 enzymes also showed that other P450-mediated oxidative reactions in the ecgonine and benzoic acid groups of cocaine contributed to the species-dependent biotransformation of cocaine [37]. Zeng et al. [39] used GC-MS-based metabolomics to study the effect of 10-days of heroin exposure, followed by a withdrawal of four days in a rat animal model. Analysis of the metabolites revealed that heroin administration decreased tryptophan and 5-hydroxytryptamine levels in peripheral serum, but increased tryptophan and 5-hydroxyindoleacetate in urine. Withdrawal of heroin for four days efficiently restored all metabolites to baseline, except serum myo-inositol-1-phosphate, threonate, and hydroxyproline in the urine. These biomarkers were discussed as potential indicators of heroin abuse, even when the consumer has not used heroin for some days [39].

### 3.3. Other Aspects of Drug Intoxication

The last and significant group of currently available metabolomics studies associated with drugs of abuse includes studies primarily aiming to explain drug action mechanisms and may provide a means to divulge underlying metabolic perturbations associated with drug addiction [40]. The methods selected for a metabolomics investigation have been organized and carried out by analyzing samples from human and animal models to find putative metabolite biomarkers linked to drug addiction and lifestyle choices, which can influence addiction-related outcomes. Moreover, studies using in vivo model systems should be designed with a specific dose and time to respond to the identification of early or prescient markers of an addiction profile. Adequately designed experiments using biological fluids as well as organ tissues should provide a knowledge adequate to establish non-invasive (e.g., blood or urine) corollary markers of the target organ (e.g., brain) drug-induced changes [41]. Recent findings in drug addiction research were used to analyze brain tissue from morphine-treated versus saline-treated monkeys. The researchers reported disruptions in the concentrations of myoinositol, taurine, lactic acid, phosphocholine, creatinine, N-acetyl aspartate, g-aminobutyric acid, glutamate, glutathione, methionine, and homocysteic acid in brain hippocampus and prefrontal cortex (PFC) in the morphine-treated monkeys relative to the controls [42]. The other drug that significantly stimulates specific metabolic pathways is heroin. In the above-described experiment of Zheng et al. [39], in heroin-treated rats, feeding behavior disorder, accelerated energy metabolism (due to increased activity of the tricarboxylic acid cycle) as well as an escalation of free fatty acid (FFA) metabolism were observed. These findings were consistent with the metabolic influence of exposure as well as addiction and the withdrawal of the drug [39]. In another study from the same group, increasing doses of methamphetamine were intraperitoneally injected for male Sprague–Dawley rats for five days followed by two-days of withdrawal. GC-MS metabolomics analysis of collected serum and urine samples showed disrupted energy metabolism during methamphetamine administration including increased fatty acid beta-oxidation, accelerated tricarboxylic acid activity as well as a visible reduction in branched-chain amino acids, most of which resolved itself during the withdrawal period [36]. Another metabolomics study used MS to assess the metabolome of urine and plasma samples from methamphetamine-, morphine-, and cocaine-addicted male Sprague–Dawley rats. The levels of L-tryptophan, cystine, lactose, spermidine, 3-hydroxybutyric acid, and stearic acid were altered depending on the type of samples and drugs. These differences might help explain, at least partly, the different actions of specific drugs on the brain reward circuitry, and prove that metabolomics may be useful in predicting the extent or mechanism of drug addiction [43]. The LC-MS-based metabolomics was performed in rat and mouse urine samples to compare cocaine metabolism. Afterward, treating the rodents with the same dose of cocaine, the researchers established that, even though benzoylecgonine levels were similar, metabolites from the oxidative metabolism of cocaine such as hydroxybenzoylecgonine and N-hydroxybenzoylnorecgonine were notably higher in rats compared with mice. These results were impressive because they indicated species-specific changes in cocaine metabolism, and would be applicable when translating animal studies to humans [37]. Besides, in a neuronal metabolomics study in rats, ambient pressure ion mobility MS was used to estimate metabolic perturbations following cocaine exposure. The results displayed the cocaine effects on glucose and amine metabolites in different anatomical areas of rat striatum, PFC, and nucleus accumbens. The observed metabolome diversity in treated rats was specific to the brain region, revealing that cocaine administration had the most significant effect on glycolysis metabolism in the thalamus [44].

Detailed information about metabolites identified as the potential biomarkers of drug intoxication/abuse together with the type of material studied and analytical method used is summarized in Table 1 (human studies) and Table 2 (animal studies).

## 4. Applications of Metabolomics in Forensic Medicine

Many biochemical changes continue occurring in the body after death because of the modifications of enzymatic reactions, cellular autolysis and putrefaction, lack of circulating oxygen, and the cessation of synthetic pathways. These variations mean that metabolomics can be used to investigate the metabolic changes that occur following death. One of the most incredible goals in forensic medicine is the estimation of the time since death.

### 4.1. Estimation of the Time Since Death

Determining the time since death (PMI) is essential because having a time frame can help to identify the human remains and investigate the possible causes of death [45]. On the other hand, knowing when the death occurred may help clarify an end’s circumstances and assess any potential information made by suspects [46]. Due to these remarks, increasing numbers of researchers are trying to find a more accurate way of estimating the time since death including compounds involved in metabolism (metabolites).

Although studies on post-mortal metabolic deterioration have a relatively modest history in forensic medicine, various metabolites have been suggested as possible PMI markers over the years. In 1984, Harada et al. [47] investigated metabolite changes in rat liver, spleen, brain, heart, and the dorsal muscle and indicated that lactate and pyruvate could be related to PMI in heart and muscle tissue [47]. The researchers also concluded that this relationship varies with the cause of death. Two years later, Sparks et al. [48] presented a linear relationship between 3-methoxytyramine (3-MT) and PMI in the dorsal putamen [48]. Postmortem changes in brain metabolites have been widely studied by different teams [49,50,51]. Free trimethylammonium (fTMA) has been suggested as a suitable marker in all studies. In 2002, Ith et al. [52] conducted the study concentrated on decomposition of the brain and found similarities between the deterioration of human and sheep brains [52]. In 2005, Banaschak et al. [53] investigated the post-mortal changes of metabolites in pig brains and identified fTMA as well as creatinine, lactate, acetate, succinate, and alanine [53]. Similar results were obtained by Scheuer et al. [49], who published, in the same year, a 1H-NMR-based study showing a high correlation between the PMI and the level of acetate, alanine, and fTMA in a sheep model [49].

More recent studies have proposed polysaccharides, steroids, amino acids, and others as potential biomarkers allowing for the estimation of the time since death [54]. The correlation between PMI and the ATPs breakdown products in rat brain, kidney, and spleen were also described [55]. At the same time, Donaldson and Lamont [56] observed changes in postmortem blood. They suggested the nitrogen-containing metabolites (ammonia, hypoxanthine, and uric acid), lactic acid, formic acid, and NADH as potential small molecule PMI markers [56]. The researchers pointed out that postmortem changes in the above-mentioned metabolites are associated with anaerobic metabolism, the absence of the cellular respiration, and are attributed to bacterial putrefaction (increase in formic acid). The next study of Donaldson and Lamont [57] provided a comprehensive overview of the metabolic changes in blood after death [57]. The authors detected postmortem changes of 66 metabolites and found that 26 of them (including 18 amino acids) were seen to have the potential to estimate the time since death. Changes to such a significant number of amino acids may depend on postmortem catabolism and putrefaction, dramatic decrease in protein synthesis before ceasing entirely, and specific synthesis of amino-acids (essential and non-essential) by mammals. Modifications in the metabolome in relation to the PMI have also been described by Sato et al. [58]. The authors performed a GC-MS/MS-based metabolic profiling of a suffocated rat model after a particular time after death. In this study, 25 (eighteen amino acids, five sugars, a carboxylic acid and a phosphate) out of 70 significant metabolites had a statistically strong correlation with PMI.

**Figure 1:**
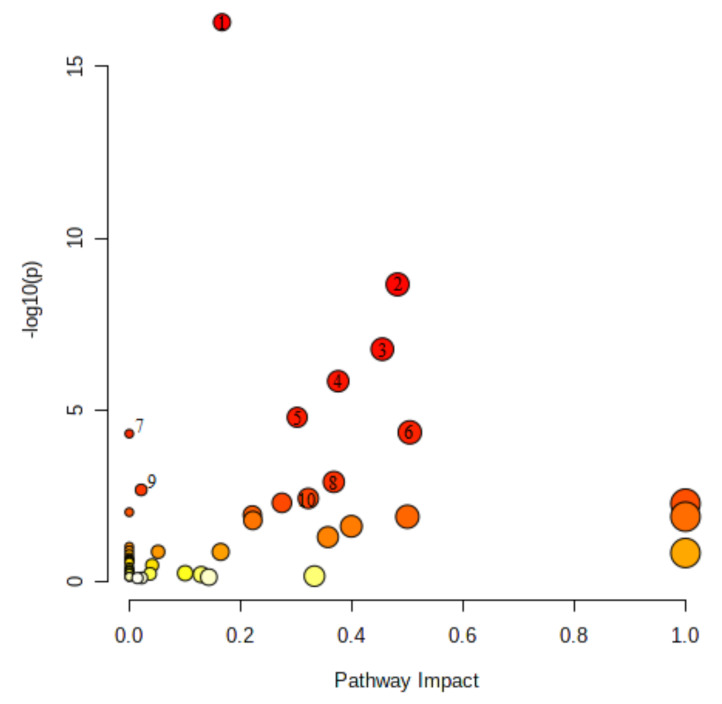
Metabolic pathway analysis performed for significant metabolites reported in rat plasma samples. Metabolic pathway analysis was carried out with MetaboAnalyst 5.0 software. Ten most significant pathways are marked with the numbers: (1) aminoacyl-tRNA biosynthesis, (2) alanine, aspartate and glutamate metabolism, (3) glyoxylate and dicarboxylate metabolism, (4) arginine biosynthesis, (5) citrate cycle (TCA cycle), (6) glycine, serine, and threonine metabolism, (7) valine, leucine and isoleucine biosynthesis, (8) glutathione metabolism, (9) pantothenate and CoA biosynthesis, and (10) pyruvate metabolism.

To give an overview of the metabolic pathways to which metabolites significantly related to PMI belong, an inter-study pathway analysis was performed for rat plasma samples using MetaboAnalyst 5.0 (Figure 1 and Table 3). In total, 46 metabolites could be assigned to 48 metabolic pathways. As seen in Figure 1, aminoacyl-tRNA biosynthesis as well as the alanine, aspartate, and glutamate metabolism were the most significantly affected metabolic pathways. The value of other pathways is also represented in Figure 1, where 10 of the most significant pathways are marked. The complete list of metabolic pathways involving metabolites identified in rat plasma samples is presented in Table 3. The table includes the number of metabolites present in the pathway and detected in rat plasma as well as the results of pathway analysis (*p* value and pathway impact value).

The newest study (NMR metabolic profiling with multivariate statistics) showed that the spleen (75 significantly changing metabolites), kidney (70), and heart (65) were the organs that displayed the largest metabolic modulations after death [59]. The metabolites that were more often associated with postmortem metabolic fluctuations included taurine, niacinamide, phenylalanine, tyrosine, glycerol, xanthine, and lactate. For example, lactate increases when small amounts of oxygen are available. The anaerobic glycolysis reserves direct pyruvate to lactate production instead of entering the Krebs cycle under normal aerobic conditions. Other metabolites such as tyrosine or phenylalanine may result from proteolysis, but have also been associated with microbial activity in living organisms [60,61]. Dai et al. [62] carried out a GC-MS metabolomics analysis of blood plasma obtained from rats after acute dichlorvos (DDVP) poisoning and selected 39 metabolites as potential markers, allowing an estimation of PMI. They also used the combination of 23 metabolites to establish support vector regression (SVR) models to investigate the PMI. The findings demonstrated the massive potential of GC-MS-based metabolomics combined with the SVR model in determining the PMI [62]. The weak point of estimation time since death by postmortem metabolic profiling could be associated with the fact that it differs widely among various causes of death [62,63]. Additionally, the concise (less than 1 s) half-lives of metabolic reactions in an organism mean that fast and varied changes in the process of dying might alter numerous, often indirectly death-related, metabolites producing misleading interpretations of the situation [64].

### 4.2. Application of Metabolomics in Post-Mortem Diagnosis

Medico-legal autopsy and subsequent postmortem investigations such as toxicology, microbiology, and histological examination allowed the determination of the cause of death in about 90–95% of all cases [65,66]. In other unclear circumstances, the cause and mechanism of death can be explained by a novel research method that can be described as “metabolomics autopsy”, showing undiscovered pathophysiological mechanisms in different death causes. Metabolic profiling of post-mortal biological specimens may explain more deeply the pathogenesis of fatal disorders like cardiovascular disease [67], fatal cancers [68,69], diabetes mellitus [70], or traumatic brain injury [71]. On the other hand, important disturbances in selected metabolic pathways leading to changes in the concentration of the indicated metabolites may constitute a significant extension of the postmortem diagnosis, especially associated with stages of the disease. Changes visible in the metabolic pathways occurring with the disorder’s progression may be useful in court proceedings, not only in determining the PMI, but also in determining the duration of the changes or confronting the circumstances of death with the explanations of witnesses and suspects.

The new generation of biomarkers (metabolomics ones) that can supply information on pathophysiological processes in the deceased can be found in various biological specimens including blood, serum, urine, vitreous humor, or cerebrospinal fluid. The appropriate selection of the specimens or tissues for the analysis may be crucial for obtaining the desired metabolites present in a biological sample. For example, Bohnert et al. [72] emphasize that cerebrospinal fluid can be primarily used for biochemical investigations in routine work because of being less prone to autolysis (compared with cadaveric blood or serum) connected to its protected anatomical location inside the skull and the spinal canal [72]. The beneficial effect of the tested specimens’ anatomical isolation was also discussed by Locci et al. [73] and Zelenstsova et al. [74]. Both teams indicated that anatomically isolated eye fluids: vitreous humor and aqueous humor have advantages over blood for metabolomics analysis. This means that the specimens above-mentioned are a desirable material for forensic research.

### 4.3. Metabolomics Aspects of Diseases Responsible for Global Mortality

Research carried out in recent years has allowed for the identification of many metabolomics changes in numerous, often fatal, diseases. A detailed description of all the abnormalities visible in so many illnesses is not possible in one study. However, it is possible to present a few examples of metabolomics changes in selected disorders.

One of the most leading causes of morbidity and mortality worldwide is cerebral ischemic disease, a common form of stroke. Cerebral ischemia is the fifth leading cause of death [75], and it is caused by the blockage of a blood vessel connected with a thrombus or embolus [76]. During brain ischemia, part of the brain lacks oxygen and nutrients, becoming damaged through induction of metabolic and cellular disturbances. As time is a decisive factor in diagnosing and treating cerebral ischemia, there are no queries that time-dependent pathophysiological mechanisms of cerebral ischemia occur through several sequential steps [77]. The first is a result of reduced blood flow and depletion of oxygen and nutrient delivery to brain tissue. The second is due to energy depletion, which leads to excitotoxicity and peri-infarct depolarization within hours. The last one is connected to proinflammatory cytokine generation by injured brain cells, recruit macrophages and monocytes to the ischemic penumbra, and trigger brain inflammation and oxidative stress within days. The inflammatory condition and reactive oxygen species trigger necrosis and apoptosis of brain cells through mitochondrial and DNA damage for many days and weeks.

Several studies have investigated metabolic changes in acute ischemic strokes of varying severities. For example, in the SPOTRIAS (Specialized Programs of Translational Research in Acute Stroke Network) biomarker study, serum metabolites in the acute phase were measured [78]. The other study, prepared by Liu et al. [79], evaluated metabolic features of the serum of ischemic stroke patients with significant deficits (NIHSS ≥ 6) compared to healthy controls [79]. These projects analyzed different ranges of metabolites, but both found that glycine, isoleucine, and lysine, which are positively related to inflammation, were present in low levels compared to the healthy control subjects. The oxidative stress visible in cerebral ischemia is caused by excessive oxygen derivatives and metabolic dysfunction [80]. In cerebral ischemia, oxygen depletion leads to anaerobic glycolysis in cells, generating lactate as a final product, making the cytosolic environment acidic [81]. Excessive protons convert oxygen into hydrogen peroxide and reactive hydroxyl radicals. As a reflection of these metabolic changes in the brain, increased formate, glycolate, lactate, and pyruvate have been reported in the plasma and urine of cerebral ischemia patients [82].

Another example of critical disorders, which is the leading cause of morbidity and mortality worldwide, is coronary artery disease, paired with myocardial infarction—a common manifestation of this disease [83]. Current markers for myocardial infarction (i.e., creatine kinase-MB and cardiac troponin T and I) can be used in postmortem examination [84] but are not reliably detected until 4–6 h post-myocardial injury [85]. Postmortem diagnosis of an early-stage heart attack is difficult because gross and microscopic examinations do not reveal the damaged heart’s characteristic changes. This means that new biomarkers are needed to facilitate interventions, preventing the disease progression and its complications including death, and allowing for better clinical and postmortem diagnosis, especially of the early stage of myocardial infarction.

In myocardial infarction, the primary recognized altered metabolic pathways are mainly referred to as oxidative stress and ischemia-induced alterations in energy metabolism, amino acids metabolism, fatty acid oxidation, anaerobic glycolysis, urea cycle, and pathways linked to endogenous gasotransmitters. Ischemic stress is also associated with elevated hydrocortisone levels, which can often blunt insulin sensitivity. Insulin resistance that develops in adipose tissue results in the altered insulin signaling cascade ability to store triglycerides. This will induce lipolysis and uncontrolled release of FFA and glycerol [86,87,88]. The metabolic alterations in myocardial energy production also affect other crucial metabolic pathways [89]. The decline in glucose oxidation during ischemia requires the rapid acceleration in pyruvate conversion to lactate to regenerate NAD+ under oxygen limiting conditions, which is needed to sustain glycolysis [90]. Increased reliance on the anaerobic myocardial metabolism occurs, which increases lactic acid release out of cardiac myocytes for maintenance of ATP levels. The elevated levels of ketone bodies are another common metabolic area that could also stress-energy metabolism. Both β-hydroxybutyrate and acetone are ketone bodies that are mainly synthesized from the oxidation of fatty acids and are known for their roles in glucose and lipid metabolism [91]. The next interesting potential biomarker that signals for either hyperglycemia or dysregulation of fatty acids metabolism is α-hydroxyisobutyric acid. The study of Farag et al. [92] showed an increase of α-hydroxyisobutyric acid blood level in individuals with myocardial infarction, arising from ineffective fatty acids oxidation [92].

### 4.4. Estimation of the Mortality Risk

Another exciting application of metabolomics, indirectly related to legal medicine, is the early identification of persons at high, short-term risk of death. Fischer et al. [93] used a high-throughput and well standardized nuclear magnetic resonance (NMR) platform. They identified metabolic markers (i.e., glycoprotein, albumin, citrate, acetyls (GlycA), and mean diameter for very-low-density lipoprotein (VLDL) particles) that are independently associated with all-cause and cause-specific (cardiovascular disease and cancer) mortality [93,94,95]. Biomarker associations with cardiovascular, nonvascular, and cancer mortality suggest novel systemic connectivities across seemingly disparate morbidities. A similar study of Deelen et al. [96], in which high-throughput metabolomics was performed, showed a group of fourteen biomarkers independently associated with all-cause mortality. Proposed biomarkers were connected with males and females and across age strata, and represented rather a general health up to the highest ages than specific disease-related death causes [96].

## 5. Conclusions

Metabolomics studies possess a high potential for the detection of biomarkers indicating drug abuse. Changes noticed so far on the endogenous level currently appear relatively small, partly unspecific, and might be insufficient on the number of markers to indisputably prove drug intake. It is also an interesting approach in xenometabolomics—particularly for rare metabolic pathways.

The metabolomics area can explain the new pathophysiological mechanism in different death causes, has excellent potential for estimating PMI in forensic investigations, and for estimating the risk of mortality.

Nevertheless, more studies are strongly needed to explore the potential of metabolomics in drug abuse analyses and other medico-legal fields.

## Figures and Tables

**Table 1:** Summary of studies using metabolomic analysis to detect metabolites as the potential biomarkers of drug intoxication/abuse in human samples.

Drug	Analytical Method	Sample Type	Potential Biomarkers of Drug Intoxication/Abuse	Ref
Cannabinoids (Synthetic)	LC-QTOF-MS	saliva	scopoletin and 2-hydroxyethyl dodecylamine	[35]
Crack	1H-NMR	serum	Lactate, carnitine, histidine, tyrosine	[38]
GHB	LC-QTOF-MS	urine	GHB carnitine, GHB glycine, and GHB glutamate	[29]
MDMA (ecstasy)	LC-QTOF-MS	blood	adenosine monophosphate, adenosine, inosine, S-adenosyl-L-homocysteine, tryptophan, thiomorpholine 3-carboxylate, LysoPC (16:0), LysoPC (17:0), LysoPC (18:1)	[34]
Valproic acid	LC-MS	blood	3-hydroxy-4-en-VPA	[31]

LC-QTOF-MS–liquid chromatography coupled with quadrupole time-of-flight mass spectrometry, 1H-NMR–proton nuclear magnetic resonance, LC-MS-liquid chromatography coupled with mass spectrometry.

**Table 2:** Summary of studies using metabolomic analysis to detect metabolites as the potential biomarkers of drug intoxication/abuse in animal samples.

Drug	Analytical Method	Organism	Sample Type	Potential Biomarkers of Drug Intoxication/Abuse	Ref
Cocaine	GC-MS	rat	plasma	threonine, cystine, spermidine	[43]
	IM-MS		brain tissue	5-hydroxyindoleacetic acid, glucose, norepinephrine, serotonin	[44]
Methamphetamine	CE-MS	rat	urine	fructose 1,6-bisphosphate, fumarate, glucose 6-phosphate	[33]
	GC-TOF-MS	rat	urine	5-oxoproline, saccharic acid, uracil, fumarate,3-hydroxybutyrate, adipic acid	
			plasma	glucose and 3-hydroxybutyrate	
	GC-MS	rat	serum	creatinine, citrate, 2-ketoglutarate	[36]
			urine	lactate	
				lactose, spermidine, stearic acid	[43]
			plasma	n-propylamine, lauric acid	
Morphine	GC-MS	rat	urine	2-ketoglutaric acid, fumaric acid, malic acid, L-threonine, glutamic acid, isoleucine, L-valine, L-aspartic acid, oxamic acid, 2-aminoethanol, indoxyl sulfate, creatinine	[43]
			plasma	L-tryptophan, 3-hydroxybutyric acid, cystine, n-propylamine	
	1H-NMR	monkey	brain tissue	myoinositol, taurine, lactic acid, phosphocholine, creatinine, N-acetyl aspartate, g-aminobutyric acid, glutamate, glutathione, methionine, homocysteic acid	[42]
Heroin	GC-MS	rat	serum	myo-inositol-1-phosphate, threonate	[39]
			urine	hydroxyproline	

GC-MS-gas chromatography coupled with mass spectrometry, IM-MS–ion mobility mass spectrometry, CE-MS–capillary electrophoresis coupled with mass spectrometry, GC-TOF-MS-gas chromatography coupled with time-of-flight mass spectrometry.

**Table 3:** Metabolic pathways corresponding to metabolites identified in rat plasma samples.

Pathway	No. of Metabolites in the Pathway	No. of Metabolites Detected in Plasma	*p*-Value	Pathway Impact
Aminoacyl-tRNA biosynthesis	48	18	5.16 × 10^−17^	0.17
Alanine, aspartate and glutamate metabolism	28	10	2.19 × 10^−9^	0.48
Glyoxylate and dicarboxylate metabolism	32	9	1.69 × 10^−7^	0.46
Arginine biosynthesis	14	6	1.44 × 10^−6^	0.38
Citrate cycle (TCA cycle)	20	6	1.62 × 10^−5^	0.30
Glycine, serine and threonine metabolism	34	7	4.43 × 10^−5^	0.50
Valine, leucine and isoleucine biosynthesis	8	4	4.85 × 10^−05^	0.00
Glutathione metabolism	28	5	0.001227	0.37
Pantothenate and CoA biosynthesis	19	4	0.002097	0.02
Pyruvate metabolism	22	4	0.0037003	0.32
Arginine and proline metabolism	38	5	0.0049851	0.27
Phenylalanine, tyrosine and tryptophan biosynthesis	4	2	0.0052478	1.00
Butanoate metabolism	15	3	0.009333	0.00
Histidine metabolism	16	3	0.011244	0.22
D-Glutamine and D-glutamate metabolism	6	2	0.012616	0.50
Nitrogen metabolism	6	2	0.012616	1.00
Cysteine and methionine metabolism	33	4	0.016192	0.22
beta-Alanine metabolism	21	3	0.024004	0.40
Phenylalanine metabolism	12	2	0.049413	0.36
Biosynthesis of unsaturated fatty acids	36	3	0.093951	0.00
Valine, leucine and isoleucine degradation	40	3	0.11962	0.00
Pentose phosphate pathway	21	2	0.13242	0.05
Tyrosine metabolism	42	3	0.13335	0.16
Linoleic acid metabolism	5	1	0.14358	1.00
Propanoate metabolism	23	2	0.15364	0.00
Thiamine metabolism	7	1	0.19519	0.00
Taurine and hypotaurine metabolism	8	1	0.21984	0.00
Porphyrin and chlorophyll metabolism	30	2	0.23186	0.00
Ubiquinone and other terpenoid-quinone biosynthesis	9	1	0.24374	0.00
Ascorbate and aldarate metabolism	10	1	0.26694	0.00
Biotin metabolism	10	1	0.26694	0.00
Purine metabolism	66	3	0.32642	0.04
Nicotinate and nicotinamide metabolism	15	1	0.37285	0.00
Pentose and glucuronate interconversions	18	1	0.42905	0.00
Selenocompound metabolism	20	1	0.46375	0.00
Sphingolipid metabolism	21	1	0.48032	0.00
Lysine degradation	25	1	0.54172	0.00
Glycolysis / Gluconeogenesis	26	1	0.55592	0.10
Galactose metabolism	27	1	0.5697	0.00
Phosphatidylinositol signaling system	28	1	0.58305	0.04
Inositol phosphate metabolism	30	1	0.60856	0.13
Arachidonic acid metabolism	36	1	0.67626	0.33
Fatty acid elongation	39	1	0.70568	0.00
Fatty acid degradation	39	1	0.70568	0.00
Pyrimidine metabolism	39	1	0.70568	0.00
Tryptophan metabolism	41	1	0.72381	0.14
Primary bile acid biosynthesis	46	1	0.76451	0.02
Fatty acid biosynthesis	47	1	0.77191	0.01

Pathway impact values and *p*-values were obtained from metabolic pathway analysis performed with MetaboAnalyst 5.0 software.

## Data Availability

Data sharing is not applicable to this article.
